# Prevalence of Keratoconus in a Population-Based Study in Syria

**DOI:** 10.1155/2022/6064533

**Published:** 2022-06-23

**Authors:** Abdelrahman Salman, Taym Darwish, Marwan Ghabra, Obeda Kailani, Yusra Haddeh, Mohammad Askar, Ammar Ali, Ali Ali, Sara Alhassan

**Affiliations:** ^1^Department of Ophthalmology, Tishreen University, Latakia, Syria; ^2^Whipps Cross University Hospital, Leytonstone, London, UK; ^3^Department of Ophthalmology, King's College Hospital NHS Foundation Trust, London, UK; ^4^Department of Ophthalmology, Damascus University, Damascus, Syria; ^5^Faculty of Health Sciences, American University of Beirut, Beirut, Lebanon

## Abstract

**Aim:**

To determine the prevalence and associations of keratoconus (KC) in a university student population in Syria.

**Methods:**

A prospective multicentre cross-sectional cohort study was conducted at two universities in Syria. Student volunteers were recruited from Tishreen University (Latakia governorate) and Damascus University (Damascus governorate). All participants underwent a comprehensive ocular examination. Placido/Scheimpflug-based corneal imaging using the Sirius (CSO, Florence. Italy), and a questionnaire to evaluate the baseline characteristics and medical history, as well as to highlight possible risk factors of KC. Univariate and bivariate analyses were performed.

**Results:**

The estimated prevalence of KC among all subjects was 1.43% (*n* = 12). A strong association between eye rubbing and keratoconus was found (OR 9.33, 95% CI 2.94–29.63, *P* < 0.001). Damascus University participants had a higher prevalence of KC than Tishreen University. However, the difference was not statistically significant.

**Conclusion:**

The prevalence of keratoconus in this Syrian student population was 1.43%. The results of this study demonstrate a high prevalence of keratoconus in the study population. Early detection of keratoconus through screening may yield benefits in preventing devastating sequelae of KC in populations with a high prevalence.

## 1. Introduction

Keratoconus (KC) is an ectatic corneal disease characterised by progressive thinning of the central and paracentral portion of the cornea that results in corneal protrusion, irregular astigmatism, and visual impairment [1, 2].

Although the onset of KC peaks around the age of puberty, progression may continue to the third or fourth decade of life. Progression rarely continues following the fourth decade of life [3, 4]. The incidence of KC varies widely depending upon the geographical location, as countries with hot climate such as India, Lebanon, Iran, and Australia have a higher incidence than countries with cooler climates such as Russia and Denmark [5–10]. The annual incidence of KC ranges between 50–230 cases per 100,000 population [11]. A recent study from Denmark has shown an increase in the incidence of KC at a rate of 2- to 3-fold, from 1.24 per 100,000 person-years in 2003 to 3.8 per 100,000 in 2011 [10]. A higher prevalence of KC has been found in patients with a family history of KC and eye rubbing and in patients with a history of parental consanguinity [12].

Screening of KC is of particular importance, as earlier detection and management are essential to avoid the need for corneal transplantation, preserving visual performance. This study aimed to evaluate the prevalence of KC among university students in Syria and to establish possible risk factors of the disease.

## 2. Materials and Methods

A prospective cross-sectional multicenter study was conducted to assess the prevalence of KC and suspect KC among students in Tishreen University (Latakia governorate) and Damascus University (Damascus governorate). This study was carried out between September 2019 and March 2020 in Tishreen University and between September 2019 and November 2019 in Damascus University. Participants were recruited through a notice posted on bulletin boards in both universities. This notice included a short explanation of the signs and symptoms of the disease and that in the early stages those signs may pass unnoticed. All subjects included in this study were of Syrian-Arab ethnicity. Any participants with a history of refractive surgery were excluded from the study. Participants with nonectatic corneal pathology were excluded. Informed consent was obtained from all study participants.

This study was approved by the Research Committees of Tishreen and Damascus universities in accordance with the tenets of the Declaration of Helsinki.

### 2.1. Screening Protocol

All participants underwent a comprehensive evaluation which included the following:

1. An anonymous self-administered demographics questionnaire, history of contact lens wear, family history of KC, consanguinity, and eye rubbing. Subjects were considered as eye rubbers if they had an eye rubbing frequency of at least once a day over the past year.
2. Autorefractor keratometry (SEIKO CO, GR-3500KA, Japan), visual acuity testing, and slit-lamp examination were conducted to detect the signs of KC including apical scar, corneal thinning, Fleischer rings, and Vogt's striae
3. A dilated retinoscopic examination was conducted to detect a scissoring reflex and the Charleaux oil droplet sign
4. Placido/Sheimpflug-based corneal imaging (Sirius, Costruzione Strumenti Oftalmici, Florence, Italy). The same software version Phoenix *v*. 2.6. was used at both sites. Measurements were obtained with the eye aligned to the visual axis. Participants were asked to blink before each image and only images with an acquisition registered as “OK” according to the examination standard specifications were included. Prior to evaluation, contact lens wearers were asked to discontinue use for a period of 1 week and 3 weeks, in soft and rigid contact lens wearers, respectively.

The Sirius uses a neural network-based system to classify eyes into normal, suspect keratoconus, and keratoconus compatible. The accuracy of the Sirius classification algorithm in detecting KC and suspect KC is described elsewhere [13]. Combined assessment with Sirius and Placido-based corneal imaging provided additional assessment parameters of both anterior and posterior cornea measures and a full corneal thickness map.

All participants were examined by ophthalmologists (AA in Tishreen University and MA in Damascus University). Following examination, the corneal topographic parameters (sagittal and tangential maps, mean and maximum anterior keratometry, maximum anterior elevation, posterior maximum elevation, symmetry index front, symmetry index back, corneal thickness map, central corneal thickness, and minimum corneal thickness) were examined by two cornea consultants (AS and TD) who were blind to the results of the questionnaire.

The diagnosis of KC was made if there was (a) an irregular cornea determined by distorted keratometry mires or/and distortion of the retinoscopic reflex [14, 15]; or one of the following slit-lamp findings: Vogt striae, 2-mm arc of Fleisher ring, or corneal scarring consistent with KC [16], in addition to (b) a positive Sirius software indicator [13].

Diagnosis of suspect KC was confirmed if there was (a) absence of clinical (keratometric, retinoscopic, or biomicroscopic) signs of KC in either eye, (b) best-corrected visual acuity of 20/20 or better, and (c) a positive Sirius software indicator [13].

The final diagnosis of KC and suspect KC was confirmed by two experienced cornea consultants (AS and TD). Finally, the subjects were classified into the following:

1. Keratoconus: if one eye or both eyes had KC
2. Suspect KC: if both eyes were suspect or one eye was suspect and the contralateral was normal
3. Normal: if both eyes were normal

**Figure 1:**
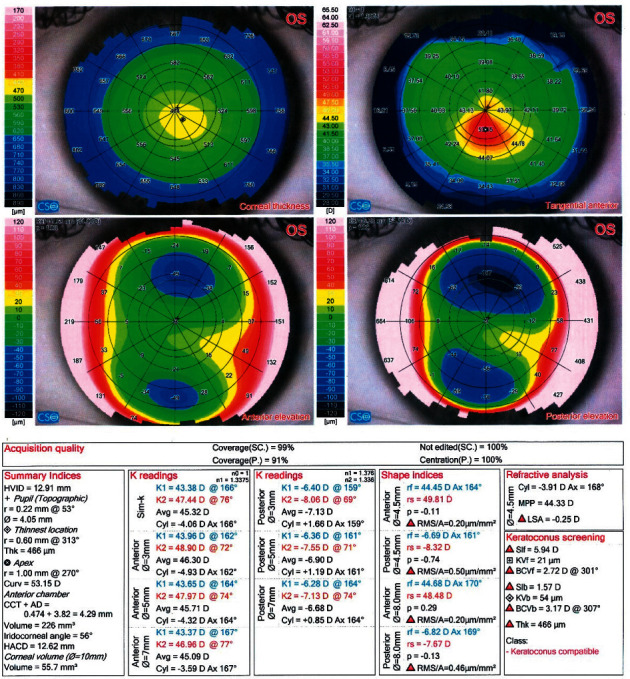
Sirius (v) 2.6 quad map of a case with keratoconus.

**Figure 2:**
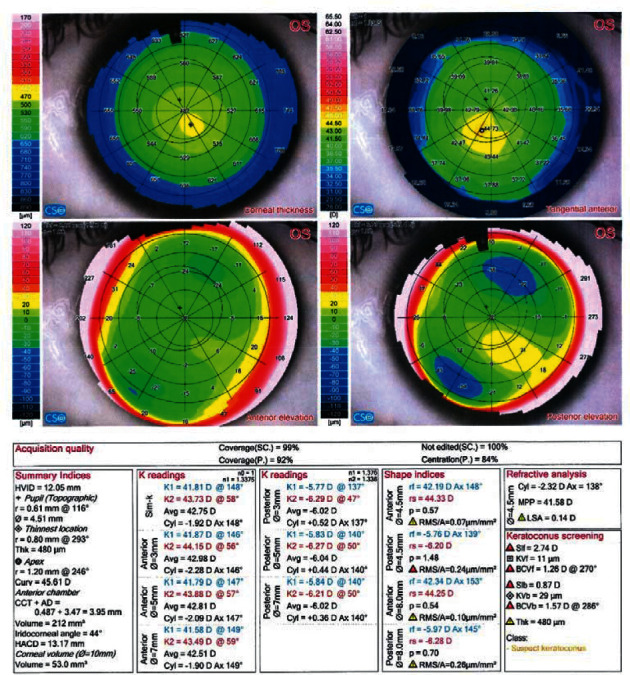
Sirius (v) 2.6 quad map of a case with a suspect keratoconus.

**Figure 3:**
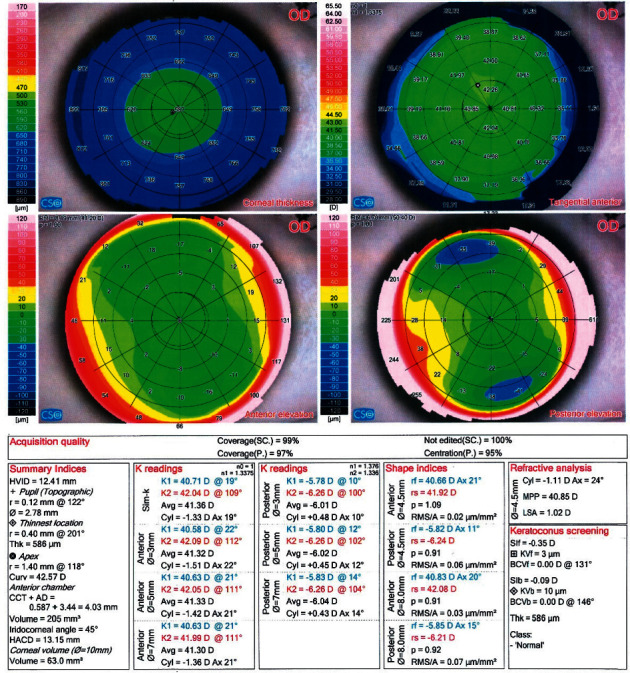
Sirius (v) 2.6 quad map of a case with a normal eye.

Figures 1–3 demonstrate representative Sirius (CSO) for KC, KC suspect, and normal eyes, respectively.

### 2.2. Statistical Analysis

Statistical analyses were conducted using the Windows SPSS software (version 25.0, SPSS, Chicago, Illinois, USA), and a *P* value of less than or equal to 0.05 was deemed statistically significant. The chi-square test was used to explore the relationship between the outcomes and the evaluation parameters.

## 3. Results

### 3.1. Demographics

A total of 881 students volunteered to participate in the study. However, 37 subjects were excluded due to incomplete datasets or did not meet the inclusion criteria. Five soft contact lens wearers participated in the analysis. However, all were excluded due to a breach of protocol in contact lens cessation prior to evaluation. A total of 839 participants met the eligibility criteria, with complete datasets and evaluation.703 (83.79%) subjects were recruited from Tishreen University with a mean age of 25.5 ± 6.1 years (range: 17–61 years) and 136 (16.20%) subjects from Damascus University with a mean age of 24.8 ± 4.4 years (range 18–40 years). The difference in the sample size between the two universities was related to the difference in the recruitment periods: 6 months in Tishreen University and two months in Damascus University. The total sample had a mean age of 25.4 ± 5.9 years, with more than half of the sample aging between 17 and 24 years (54.94%). The majority of participants were females (60.78%). Almost all the study participants had no family history of KC (99.76%), with no history of parental consanguinity (90.22%). Moreover, most of the participants were not considered eye rubbers (89.74%). While 1153 (68.71%) eyes had astigmatism and 1279 (77.29%) had myopia, only 155 (9.24%) eyes were emmetropic. Of the 767 participants who were ametropic, 753 (98.17%) participants were spectacles wearers, while 14 participants did not have any refractive error correction.

The prevalence of KC among the sample was found to be 1.43% (12 subjects) (Table 1). Of the 12 KC subjects, 10 had bilateral KC and 2 had unilateral KC (22 eyes). Although the Sirius software classifies the eyes into suspect KC and definite KC, it does not grade definite KC. Therefore, eyes diagnosed with definite KC were classified according to Amsler–Krumeich criteria where 16 KC eyes were found to be stage 1 and 6 had stage 2. None of the KC eyes revealed abnormal slit-lamp findings.

Table 2 demonstrates the tomographic characteristics of the study groups. The three outcome categories (normal, suspect, and keratoconus) were compared for their simulated keratometry values; flat keratometry (K1), steep keratometry (K2), average keratometry (K avg), and corneal pachymetric values; minimum corneal thickness (ThkMin) and central corneal thickness (CCT).

The KC group had higher simulated keratometry values and thinner pachymetric values than the normal group.

Based on the cone location, 20 eyes were characterised by a central cone, with an apex within the central 2 mm. Two eyes were noncentral KC (apex outside central 2 mm). Central KC had higher keratometric values and thinner pachymetric values than noncentral KC.

Table 3 demonstrates the basic characteristics of the study groups. Of the 12 KC subjects, 8 (66.67%) were female and 2 (16.67%) had a family history of KC. Parental first-relation consanguinity was reported in 2 (16.67%) KC subjects, while daily significant eye rubbing was reported in 6 (50%) subjects.

Table 4 demonstrates the association analysis results of the various independent factors evaluated in KC and normal subjects. The prevalence of KC was 1.51% in the 17–24-year-old group, 1.49% in the 25–32-year-old group, and 0.97% in those who were between 33 and 40 years. Although the difference was not statistically significant, the prevalence of KC was higher in females (1.56%) than in males (1.21%) (*P*=0.675). Eye rubbing was found to be significantly associated with the diagnoses of KC (OR 9.33, 95% CI 2.94–29.63, *P* < 0.001). Although not statistically significant, the prevalence of KC was higher in Damascus University (2.20%) than in Tishreen University (1.28%), (*P*=0.067). Age, sex, and parents' relation were not statistically significant and nonsignificant predictors (*P* > 0.05 for all) for KC.

## 4. Discussion

The current study assessed KC prevalence among a student population in two major universities in Syria; Tishreen University (Latakia governorate) and Damascus University (Damascus governorate). We found an overall prevalence of 1.43%, with a higher, but not statistically significant, prevalence for females.

In comparison to similar studies from the Middle East, our result exhibits a lower prevalence rate than a study conducted in Lebanon by Waked et al., who reported a prevalence rate of 3.3%, in a sample of 110 participants [6]. However, our results are in line with a study conducted in Nablus, Palestine, by Shehadeh et al., who reported a 1.5% prevalence rate [17]. Another study was conducted in the Middle East in Tehran, Iran, by Hashemi et al., where the overall prevalence rate of KC was 3.3% [18]. The prevalence rate was 0.8% in the 14–29-year-old-group and 7.5% in those who were over 60 years; this is in contrast to our findings. The prevalence rate of KC was 1.51% in subjects between 17 and 24 years, while none of the 8 subjects who were over 60 had KC. However, many factors may have attributed to the difference between our results and those of the Iranian study such as the mean age: 40.8 ± 17.1 compared to 25.4 ± 5.9 years in our study. In addition to the difference in the samples size, 839 subjects were in our study versus 263 subjects were in Hashemi study. In addition to the difference in diagnostic criteria between the two studies, our diagnostic criteria included clinical, topographic, and tomographic findings, while Hashemi screening for KC was based on topographic criteria only. In this study, only one case of the 12 keratoconic subjects was found in the 40–60-year-old age group, while none of the eight participants who were over 60 had KC. Although a nonsignificant correlation was found, our study showed that KC prevalence decreased with age.

Prakash et al. found that noncentral KC has a lesser effect on simulated keratometries and pachymetry than central KC [19]. Consistent with these findings, we found that central keratoconic eyes were associated with higher simulated keratometries and thinner pchymetries than those of noncentral KC.

Association between KC and positive family history is controversial, and data suggest ranges between 6% and 23.5% [20]. Our results are consistent with the study conducted in Saudi Arabia by Assiri et al., where 16% of KC patients had a positive family history of KC [21]. In the same trend, we found that 16.67% of KC patients had a positive parent's relation (consanguinity). The current study did not identify family history nor consanguinity as a significant risk factor for KC.

Several mechanisms have been suggested to explain the association of eye rubbing and pathogenesis of KC. Kallinikos and Efron hypothesized that persistent eye rubbing induced epithelial trauma and increased the release of interleukin (IL)-8 and other degenerative enzymes, facilitating the loss of the corneal fibroblasts, resulting in reduced biomechanical stability and ectasia [22]. Furthermore, fluctuation in intraocular pressure due to eye rubbing can traumatize the stromal keratocytes. In a study by Rabinowitz, eye rubbing was positive in 83% of 218 patients with KC [20]. In this study, eye rubbing was the strongest predictor to be associated with KC (*P* < 0.001), where six (50%) participants reported a positive eye rubbing.

Our results indicated a higher prevalence of KC at Damascus University, 2.20% compared to 1.21% at Tishreen University (Latakia city). The prevalence of KC is influenced by ethnicity and environment [23]. Ethnic differences between Latakia and Damascus could be a contributing factor, as Damascus is ethnically Aramean, while Latakia is ethnically Phoenician. However, this assumption should be taken with caution as the ethnic mix in the city does not necessarily reflect the student population who may come from neighbouring cities. Also, it is possible that environmental factors may have contributed to this difference. While Latakia is a coastal city with a Mediterranean humid climate, Damascus is located in the southwest of Syria, 80 km from the Mediterranean Sea and 750 meters above sea level, with a hot, dry, and sunny climate. The mean annual number of hours of sunshine is 3634 in Damascus [24] versus 2925 in Latakia [25]. Although the relative contribution of these factors is currently unknown, a positive correlation between KC and ultraviolet (UV) exposure has been reported. Excessive exposure to UV causes exudative damage to the cornea resulting in reduced amounts of key enzymes such as aldehyde dehydrogenase class 3 (ALDH3), catalase, or superoxide dismutase that are necessary in the removal of proinflammatory reactive oxygen species (ROS) [26, 27]. The inability of the KC cornea to process ROS leads to a degradative process leading ultimately to corneal thinning and ectasia. However, these results should be taken with caution for several reasons. First, the ethnic mix in the city does not necessarily reflect the student population who may have immigrated from neighbouring districts or cities. In addition, the variability in the study sample and duration of acquisition may be a limiting factor contributing to the dissimilarity in the study samples.

There are several limitations to this study. A selection bias may have occurred since students who had a prior diagnosis of KC may have refrained from participation, due to ongoing ophthalmic follow-up, which may explain why none of the 12 participants with KC were previously diagnosed. On the contrary, the recruitment method would likely attract subjects who may be worried they have KC (visual symptoms, eye rubbing, myopic or those who have glasses, or a change in refraction). In addition, the small number of subjects with KC lowers the power when evaluating associated risk factors.

However, our results showed that the prevalence of KC among Tishreen and Damascus University students in Syria was relatively high, and among the highest in the world. Since all KC subjects were newly diagnosed in the study, we recommend regulated screening programs for KC for university students and the younger population. Furthermore, 4.64% were KC suspects and they should be followed up regularly.

To the best of our knowledge, this is the first study in Syria to assess the prevalence of KC.

## 5. Conclusion

A high prevalence of keratoconus (1.43%) was found among Tishreen and Damascus Universities students in Syria. National screening programs to detect keratoconus at its earliest stage is the key to enable early management, halt progression, and maximise visual outcome.

## Figures and Tables

**Table 1:** Summary statistics of categorical variables.

Variable		Count (*N* = 839)	Valid percent
Age	17–24	461	54.94
	25–32	267	31.82
	33–40	103	12.27
	41 and above	8	0.95
Sex	Female	510	60.78
	Male	329	39.21
University	Tishreen	703	83.79
	Damascus	136	16.20
Family history	No	837	99.76
	Yes	2	0.23
Parents relationship	No	757	90.22
	Yes	82	9.77
Eye rubbing	No	753	89.74
	Yes	86	10.25
Patient outcome	Normal	788	93.92
	Suspect	39	4.64
	Keratoconus	12	1.43

		Count (N *=* 1678)	Valid percent

Astigmatic	No	525	31.29
	Yes	1153	68.71
Myopic	No	381	22.71
	Yes	1297	77.29
Hyperopic	No	1493	88.97
	Yes	185	11.03
Emmetropic	No	1523	90.76
	Yes	155	9.24

**Table 2:** Bivariate summary statistics of the outcome (patient outcome) and continuous explanatory variables.

Variable	Mean ± SD		
	Normal (*N* = 788)	Suspect (*N* = 39)	Keratoconus (*N* = 12)
K1 (D)	42.7 ± 1.5	43.2 ± 1.2	45.1 ± 2.1
K2 (D)	43.8 ± 1.7	44.3 ± 1.3	48.6 ± 2.9
K Avg (D)	43.2 ± 1.4	43.7 ± 1.2	46.8 ± 2.4
ThKMin (um)	545.3 ± 32.8	510.5 ± 35.7	468.9 ± 34.3
CCT (um)	548.3 ± 33.6	515.0 ± 36.2	478.5 ± 34.5

*K*1 = flat keratometry; *K*2 = steep keratometry; Avg *K* = simulated average keratometry; ThkMin = minimum corneal thickness; CCT = central corneal thickness; *D* = diopter; SD = standard deviation; *N* = number.

**Table 3:** Summary statistics of the outcome (patient outcome) and categorical explanatory variables.

		Normal (*N* = 788)		Suspect (*N* = 39)		Keratoconus (*N* = 12)	
		Count	Valid %^*∗*^	Count	Valid %^*∗*^	Count	Valid %^*∗*^
Sex	Male	304	38.57	21	53.85	4	33.33
	Female	484	61.42	18	46.15	8	66.67

University	Tishreen	665	84.39	29	74.36	9	75.00
	Damascus	123	15.61	10	25.64	3	25.00

Family history	No	788	100	39	100	10	83.33
	Yes	0	0.00	0	0.00	2	16.67

Parents relationship	No	708	89.84	39	100	10	83.33
	Yes	80	10.17	0	0.00	2	16.67

Eye rubbing	No	714	90.60	33	84.62	6	50.0
	Yes	74	9.40	6	15.38	6	50.0

*N* = number; ^*∗*^column percentage calculated by variables.

**Table 4:** Results of the univariate analysis (*n* = 839).

Predictor		Non-Kc	Kc	Prevalence of Kc (%)	OR	95% CI for *β*		*P* value
						Lower bound	Upper bound	
Age	17 to 24 (reference)	454	7	1.51	1	—	—	—
	25 to 32	263	4	1.49	0.98	0.28	3.40	0.983
	33 to 40	102	1	0.97	0.63	0.07	5.22	0.674
	41 and above	8	0	0	1	—	—	—
Sex	Female (reference)	502	8	1.56	1	0.23	2.58	0.675
	Male	325	4	1.21	0.77			
Governorate	Tishreen (reference)	694	9	1.28	1	0.95	3.57	0.067
	Damascus	133	3	2.20	1.85			
Family history	No	827	10	1.19	1	—	—	—
	Yes	0	2	100	1			
Parents relationship	No	747	10	1.32	1	0.40	8.67	0.425
	Yes	80	2	2.43	1.86			
Eye rubbing	No	747	6	0.79	1	2.94	29.63	**<0.001**
	Yes	80	6	6.97	9.33			

Kc = keratoconus; OR = odds ratio; CI = confidence interval; *β* = beta coefficients; statistically significant values (*P* < 0.05). Values in bold are statistically significant.

## Data Availability

The datasets used and analyzed during the current study are available from the corresponding author (AS) upon reasonable request.
